# The Book World of Medicine and Science

**Published:** 1906-12-29

**Authors:** 


### The Book World of Medicine and Science.

Golden Rules of Medical Evidence. By Stanley B. Atkinson, M.A., M.B., B.Sc., of the Inner Temple, Barrister-at-Law; a Justice of the Peace for the County of London; Hon. Secretary of the Medico-Legal Society, London. (Bristol: John Wright and Company.)

This is a neat little work, compendious, comprehensive, suggestive, and practical. It will give timely relief to many a nervous medical witness, and bring comfort to practitioners anxious to learn about the usual fees for giving evidence in courts of law. In every page the work is full of important and necessary points. Not the least interesting part is the section which deals with the stepping-stones of medical evidence. The volume is one of the most useful of the "Golden Rules Series."

